# Trends in mortality due to aplastic anemia in the United States, 1999-2020: a center for disease control wonder analysis

**DOI:** 10.1016/j.htct.2026.108556

**Published:** 2026-11-02

**Authors:** Talha Khalid, Ibrahim Zulfiqar Ali, Saif Ali Malik, Noor Fatima, Amnah Khan, Ayman Irshad, Muhammad Sohaib, Zaima Afzaal, Hafiz Zerak Naseer, Fatima Aslam, Hamza Sohail, Sahil Jairamani

**Affiliations:** a Shifa College of Medicine, Islamabad, Pakistan; b Karachi Medical and Dental College, Karachi, Pakistan; c Amna Inayat Medical College, Lahore, Pakistan; d Shaikh Zayed Postgraduate Medical Institute, Lahore, Pakistan; e Ameer-ud-Din Medical College, Lahore, Pakistan; f Our Lady of Lourdes Hospital, Drogheda, Ireland; g Tucson Medical Center, Tucson, AZ, United States; h Bahawal Victoria Hospital, Bahawalpur, Pakistan; i Liaquat University of Medical and Health Sciences, Jamshoro, Pakistan

**Keywords:** Aplastic anemia, Mortality, Trends, CDC wonder

## Abstract

**Background:**

Acquired aplastic anemia is a rare, life-threatening hematologic disorder characterized by immune-mediated bone marrow failure. Despite therapeutic advancements that have increased survival, evaluating contemporary mortality trends in the United States remains important to inform clinical practice and health policy.

**Objectives:**

This study examined aplastic anemia-related mortality trends, demographic disparities, and geographic variation among United States adults from 1999 to 2020.

**Methods:**

Death certificate data from the Centers for Disease Control and Prevention Wide-Ranging Online Data for Epidemiologic Research database were analyzed for adults aged 25 years and older. Age-adjusted mortality rates per 100,000 persons and annual percent changes were calculated across years, sex, race, ethnicity, and geographic regions. Place of death and state-level social vulnerability index correlations were also evaluated.

**Results:**

Aplastic anemia was recorded in 107,975 deaths from 1999 to 2020. The national age-adjusted mortality rate decreased initially from 1999 to 2002, remained stable until 2015, and increased through 2020. Men consistently demonstrated higher age-adjusted mortality rates than women. Non-Hispanic Black or African American individuals had the highest mortality rates among racial groups. Mortality was higher in non-metropolitan areas and the Midwest region. Mortality varied widely across states, whereas state-level social vulnerability was not associated with mortality rates.

**Conclusions:**

Following a period of stability, aplastic anemia-related mortality has increased in the United States since 2015. Disparities persist across sex, race, urbanization, and geographical region.

## Introduction

Aplastic anemia (AA) is a serious hematologic condition characterized by the failure of the bone marrow to produce sufficient blood cells, leading to significant morbidity and mortality. Traditionally, AA has been considered universally fatal without therapy; however, advances such as immunosuppressive therapy and stem cell transplantation have significantly improved the prognosis for patients with severe AA [1,2]. Despite these advancements, the 5-year survival rate, regardless of treatment method, declines with age. While reliable data on the survival rate of AA in the United States (US) remains limited, a study conducted in Sweden showed that survival rates decreased from 90.5% in those aged 25–39, 70.7% in the 40–59 age group, and 38.1% in patients aged ≥60 [3]. A comprehensive understanding of AA mortality trends, particularly in the US, is lacking. Thus, investigating these demographics can provide key insights for effective public health initiatives and resource allocation [4].

The incidence of AA varies by region, ranging from 1.5–7 cases per million people annually, as mentioned in the Swedish study [3]. Although the disease is rare with low incidence, it carries a high fatality rate. Older age and severe disease at diagnosis are linked to lower survival rates [4]. Shifts in AA epidemiology can reflect broader trends in public health. Centers for Disease Control and Prevention Wide-Ranging Online Data for Epidemiologic Research (CDC Wonder) data could improve the understanding of health disparities, as predictors of adverse outcomes in patients with AA are often associated with age, sex, and race or ethnicity [1].

Utilizing this epidemiological data can help identify trends and guide public health strategies over time in the US. Given that AA is increasingly being recognized as a treatable condition among many patients [5,6], understanding mortality trends, particularly when broken down by specific demographic variables, is vital for assessing the effectiveness of such treatments [7,8]. To our knowledge, no study has reported AA mortality trends over two decades in the US. This study therefore analyzed CDC WONDER data from 1999 to 2020 to characterize AA-related mortality trends and disparities by demographic and geographic factors.

## Methods

### Study setting and population

This retrospective descriptive study utilized data from the CDC WONDER database, which includes death certificate information from all 50 states and the District of Columbia [9]. Mortality data related to AA were specifically extracted for adults in the US between 1999 and 2020. The data were categorized using the International Statistical Classification of Diseases and Related Health Problems, 10th Revision (ICD-10) codes, with code D61 representing AA. ICD-10 codes D61.1, D61.2, D61.3, D61.8, and D61.9 were specifically included. The code D61.0 for constitutional AA was excluded because it encompasses inherited bone marrow failure syndromes that are biologically and clinically distinct from acquired AA. This strategy enabled the study to isolate acquired AA and related non-constitutional marrow failure syndromes**.** The Multiple Cause-of-Death Public Use death certificate records were analyzed to select AA-related deaths, which were identified as those with AA reported as a contributory or underlying cause of death. Adults were defined as individuals aged 25 and older at the time of death. Since this study relied on de-identified, publicly available government data, local institutional review board (IRB) approval was not required. The study adhered to the Strengthening the Reporting of Observational Studies in Epidemiology (STROBE) guidelines.

### Data extraction

Data were extracted and analyzed based on several factors: demographics, population size, year, location of death, urban-rural classification, and region. Two independent authors extracted and analyzed the data, while a third author verified the results. Demographic factors included gender, age, and race/ethnicity, with a focus on individuals aged 25 and older. The ethnic categories examined were Hispanics/Latinos, Non-Hispanic (NH) Blacks or African Americans, NH Asians or Pacific Islanders, NH American Indians or Alaska Natives, and NH Whites. For the location of death analysis, the medical-facility category included inpatient facility, outpatient/emergency room, inpatient, dead on arrival, and ‘medical-facility status unknown’, categories available in CDC WONDER. The medical-facility category was compared with home/hospice as the main mortality-site contrast. For urban-rural classification, the National Center for Health Statistics Urban-Rural Classification Scheme was applied, dividing the population into urban (large metropolitan areas with a population ≥1 million, and medium/small metropolitan areas with a population between 50,000 and 999,999) and rural (population <50,000) counties, as defined by the 2013 U.S. Census. Regions were further classified as Northeast, Midwest, South, and West, based on Census Bureau definitions [10,11]. County-level data from the CDC Agency for Toxic Substances and Disease Registry 2020 Social Vulnerability Index (SVI) were incorporated to capture socioeconomic and geographic context [12]. County-level SVI values were aggregated to the state level using population-weighted means, and both the overall SVI percentile rank and theme-specific SVI ranks were used as state-level measures of socioeconomic vulnerability. Insurance status was not available in the CDC WONDER Multiple Cause of Death database and was therefore excluded from the analysis.

## Statistical analysis

To explore trends in AA-related mortality, crude and age-adjusted mortality rates (AAMRs) were calculated per 100,000 population from 1999 to 2020. These rates were grouped by year, sex, race/ethnicity, region, and urban-rural status, with 95% confidence intervals (95% CIs). Crude mortality rates were determined by dividing the number of AA-related deaths by the corresponding US population for each year. AAMRs were calculated by standardizing AA-related deaths to the population of 2000 [11]. To quantify national annual trends in AA-related mortality, the Joinpoint Regression Program (Joinpoint V 4.9.0.0, National Cancer Institute) was used to estimate the annual percent change (APC) in AAMRs, with 95% CIs [13,14]. This method identifies significant changes in AAMR over time by fitting log-linear regression models that account for temporal variations. APCs were considered increasing or decreasing if the slope describing the change in mortality differed significantly from zero, as determined by 2-tailed *t*-tests. A p-value of <0.05 was considered statistically significant. For the intersectionality analysis, place of death was examined across combined sex and race/ethnicity strata. Place of death was categorized into four groups: medical facility, home/hospice, nursing home or long-term care facility, and other/unknown. Differences in the distribution of place of death across strata were assessed using the chi-square test, with Cramér's V calculated as a measure of effect size.

State-level AAMR were correlated with population-weighted state-level SVI scores using both Pearson and Spearman correlation coefficients. To assess whether racial and ethnic mortality gaps were wider in states with greater socioeconomic vulnerability, Black to White AAMR differences, Black to White AAMR ratios, and the range between the highest and lowest race-and-ethnicity-specific AAMRs were calculated at the state level where sufficient data were available. Each parameter was then correlated with state-level SVI. Cells suppressed by CDC WONDER were excluded and not reconstructed.

## Results

A total of 107,975 deaths related to AA occurred in adults aged ≥25 between 1999 and 2020 (Supplemental Table 1). Of these deaths, 62.69% occurred in medical facilities, 17.89% occurred at home, 16.58% occurred at nursing home/hospice facilities, and 2.61% occurred at unknown or unclassified locations (Supplemental Table 2).

### Annual trends

The AAMR showed an overall increase from 2.51 in 1999 to 2.58 in 2020. An initial decrease from 1999 to 2002 (APC: −4.22; 95% CI: −8.03 to −0.25) was followed by a period of relative consistency until 2015 (APC: 0.08; 95% CI: −0.31 to 0.48), after which there was a significant increase until 2020 (APC: 3.11; 95% CI: 1.73 to 4.50) (Supplemental Table 1, Supplemental Table 3).

### Age-adjusted mortality rates stratified by sex

Throughout the years from 1999 to 2020, men consistently had a higher AAMR than women (overall AAMR for men: 2.81; 95% CI: 2.79 to 2.84; overall AAMR for women: 1.90; 95% CI: 1.88 to 1.91). From 1999–2001 the AAMR for men showed a decrease from 3.27 to 2.82, (APC: −6.80; 95% CI: −14.62 to 1.76), this was followed by a period of relative stability until 2015 when AAMR decreased slightly to 2.64 (APC: −0.23; 95% CI: −0.66 to 0.21), however, this was succeeded by a sharp increase from 2015 to 2020 at which point it reached 3.16 (APC: 3.08; 95% CI: 1.50 to 4.69). The AAMR for women over the study period showed a similar trend. Initially, the AAMR in 1999 was 2.04 (95% CI: 1.95 to 2.13), followed by decrease to 1.74 in 2002 (APC: −4.81; 95% CI: −7.70 to −1.82), then a less steep decline to 1.8 in 2015 (APC: 0.16; 95% CI: −0.66 to 0.21), after which it showed a significant increase to 2.18 in 2020 (APC: 3.36; 95% CI: 1.96 to 4.79) (Figure 1, Supplemental Table 3 and 4).

**Fig. 1:**
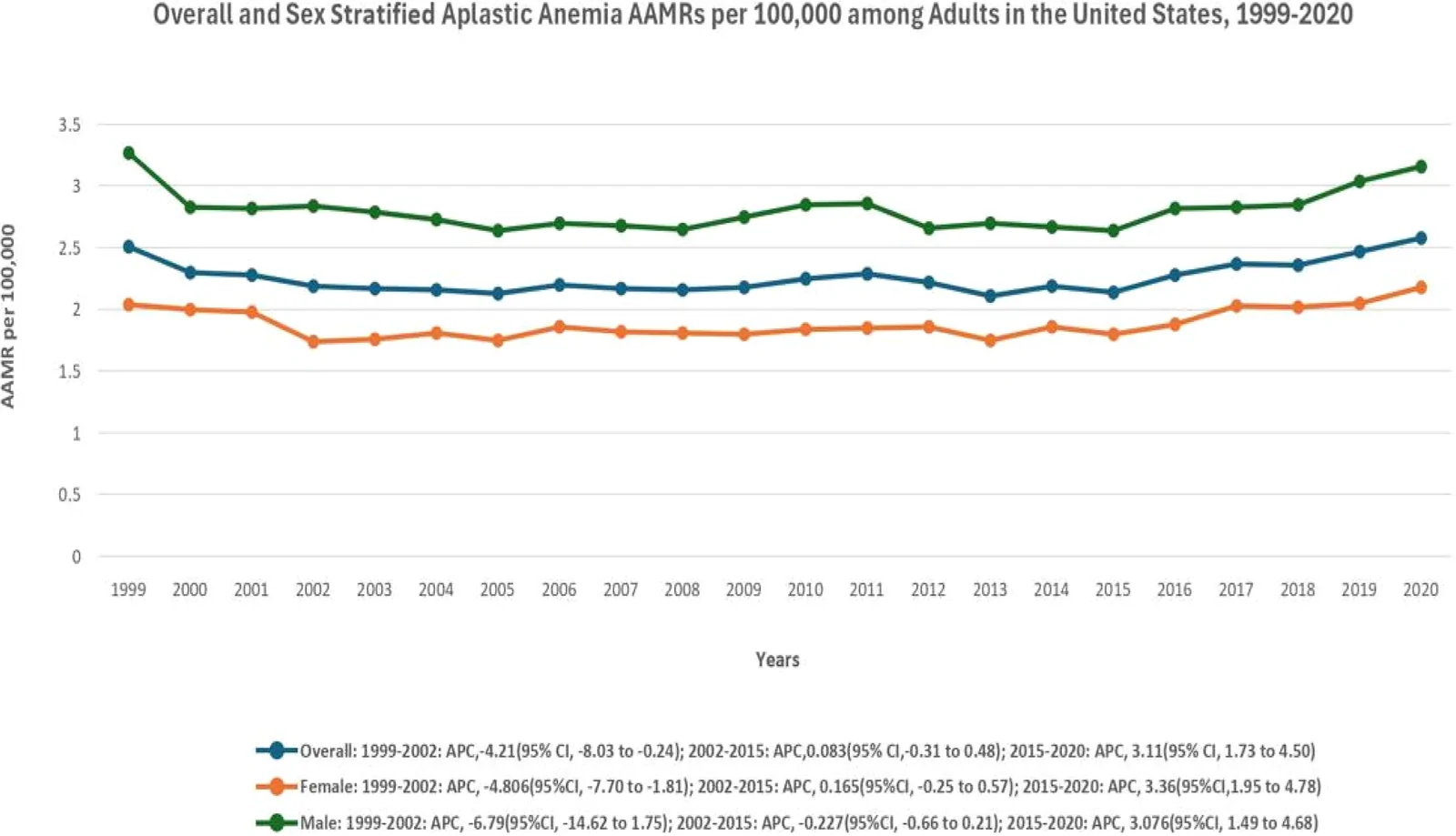
Annual trends in the age-adjusted mortality rate attributable to aplastic anemia among United States adults, overall and by sex, 1999–2020.

### Age-adjusted mortality rates stratified by race or ethnicity

From 1999 to 2020, NH Black or African Americans had the highest overall AAMRs; they were followed closely by NH whites, Hispanics or Latinos, NH American Indian or Alaska Natives, and NH Asian or Pacific Islanders (overall AAMR for NH Black or African American adults: 2.44; 95% CI: 2.39 to 2.49; NH White adults: 2.27; 95% CI: 2.26 to 2.28; Hispanic or Latino adults: 2.03; 95% CI: 1.98 to 2.07; NH American Indian or Alaska Native adults: 1.98; 95% CI: 1.82 to 2.13; NH Asian or Pacific Islander adults: 1.72; 95% CI: 1.66 to 1.78).

Examining the trends over the years, the AAMRs for NH Black or African Americans and NH Whites showed significant variability. NH Black or African Americans showed an initial decline from 1999–2016 (APC: −1.21; 95% CI: −1.72 to −0.70), followed by a substantial increase through 2020 (APC: 4.71; 95% CI: 0.50 to 9.10). NH Whites similarly showed an initial period of decrease until 2002 (APC: −4.09; 95% CI: −7.86 to −0.20); however, this was followed by a period of relative stability until 2015 (APC: 0.26; 95% CI: −0.18 to 0.71) and then increased through 2020 (APC: 3.29; 95% CI: 1.97 to 4.62). Conversely, the AAMRs of Hispanic or Latinos, NH American Indian or Alaska Native, and NH Asian or Pacific Islanders did not show any significant change from 1999–2020 (Hispanic or Latinos APC: 0.28; 95% CI: −0.07 to 0.63; NH American Indian or Alaska Native APC: 0.11; 95% CI: −1.05 to 1.29; NH Asian or Pacific Islander APC: −0.60; 95% CI: −1.30 to 0.10) (Figure 2, Supplemental Table 3 and 5).

**Fig. 2:**
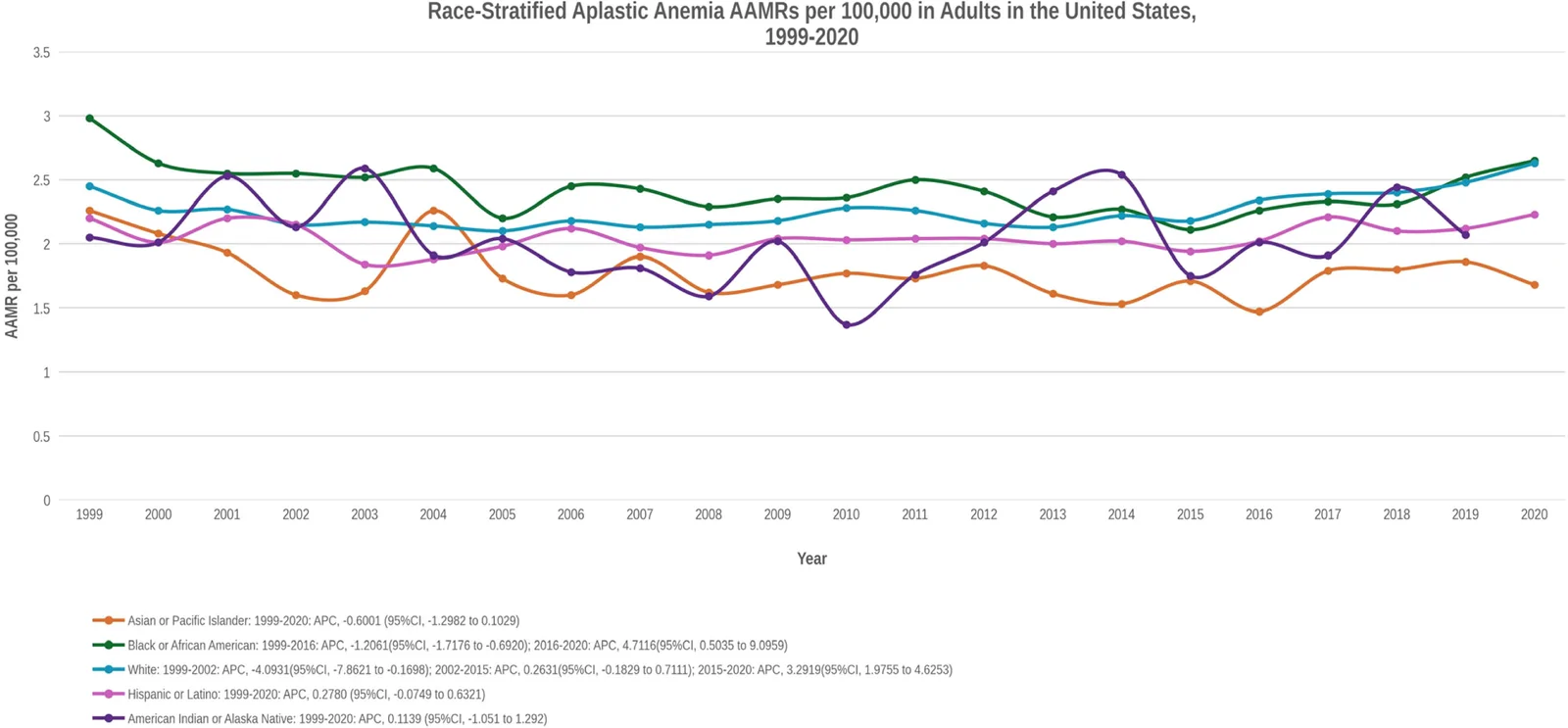
Age-adjusted mortality rates (AAMRs) due to aplastic anemia among US adults stratified by race and ethnicity, 1999–2020.

### Age-adjusted mortality rates stratified by geographical/census regions

Inspection of the trends revealed a substantial difference across states. States in the highest decile of overall AAMR included North Dakota, South Dakota, West Virginia, Tennessee, Texas, and Kentucky, whereas states in the lowest decile included New York, Massachusetts, Louisiana, New Mexico, Nevada, and Connecticut. North Dakota had the highest overall AAMR at 3.32 (95% CI: 2.99 to 3.66), more than double the AAMR of New York, which had the lowest overall rate at 1.49 (95% CI: 1.45 to 1.53) (Figure 3, Supplemental Table 6).

**Fig. 3:**
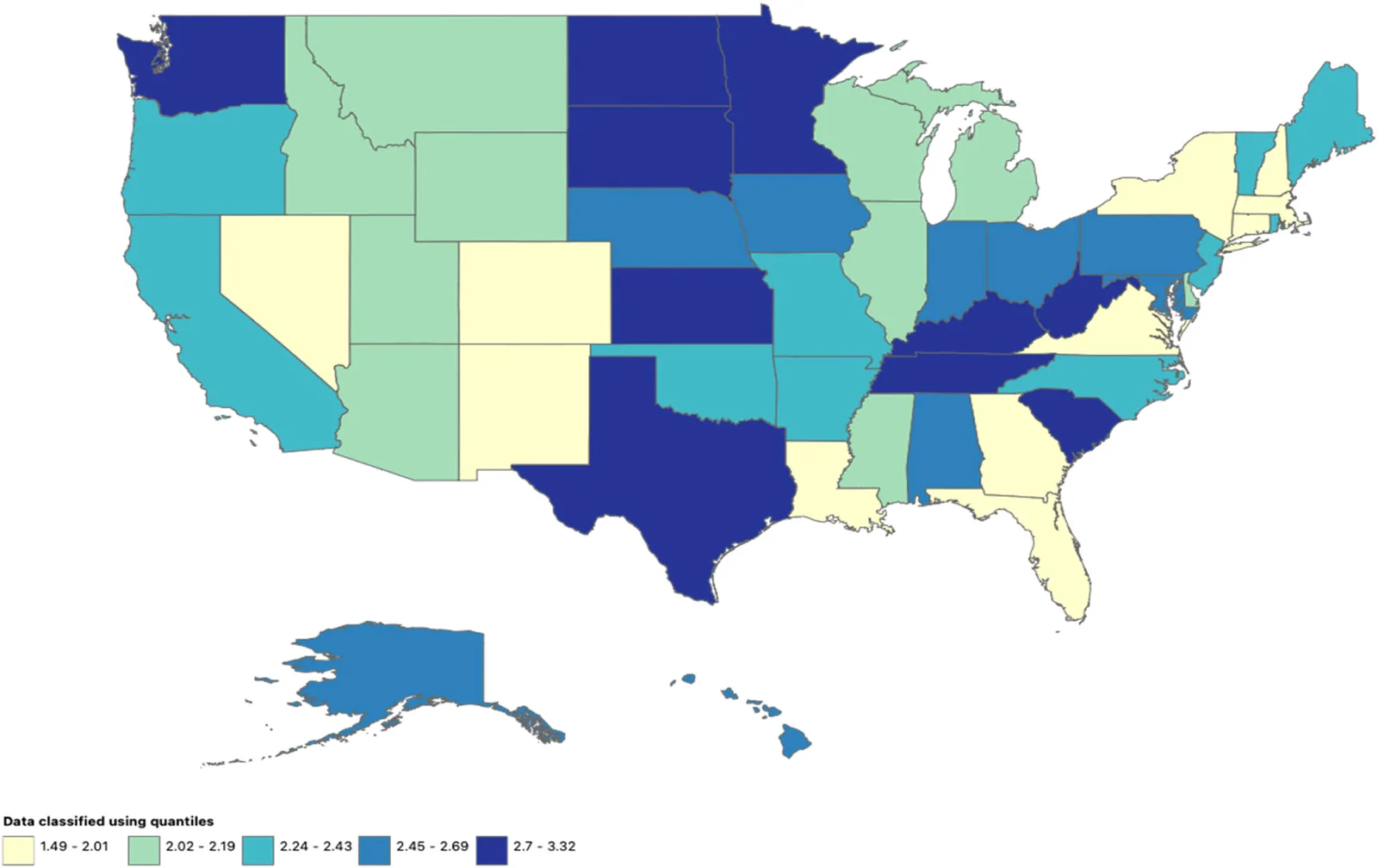
Aplastic Anemia-Related Age-adjusted mortality rates (AAMRs) per 100,000 Stratified by States in the United States, 1999–2020.

By census region, the Midwest had the highest overall AAMR (2.41; 95% CI: 2.38 to 2.44), followed by the South (2.33; 95% CI: 2.31 to 2.35), the West (2.28; 95% CI: 2.25 to 2.31), and the Northeast (1.95; 95% CI: 1.92 to 1.98) (Supplemental Tables 3 and 7).

During the study period, the Northeast experienced a significant decline in AAMR from 1999 to 2004 (APC: −4.87; 95% CI: −7.51 to −2.14), followed by relative stability from 2004 to 2020 (APC: 0.28; 95% CI: −0.21 to 0.77). The Midwest showed a significant decline from 1999 to 2015 (APC: −0.53; 95% CI: −1.06 to −0.00), followed by a significant increase from 2015 to 2020 (APC: 4.60; 95% CI: 1.85 to 7.41). The South showed a non-significant decrease from 1999 to 2002 (APC: −3.03; 95% CI: −6.53 to 0.61), stability from 2002 to 2015 (APC: 0.18; 95% CI: −0.24 to 0.60), and a significant increase from 2015 to 2020 (APC: 2.98; 95% CI: 1.66 to 4.32). The West showed a non-significant decrease from 1999 to 2001 (APC: −7.26; 95% CI: −17.39 to 4.11), relative stability from 2001 to 2014 (APC: 0.12; 95% CI: −0.55 to 0.79), and a significant increase from 2014 to 2020 (APC: 3.31; 95% CI: 1.64 to 5.00).

Non-metropolitan areas consistently had higher AAMRs than metropolitan areas (overall AAMR, 2.61; 95% CI: 2.58 to 2.65 vs 2.20; 95% CI: 2.19 to 2.22). In non-metropolitan areas, AAMR remained stable from 1999 to 2015 (APC: −0.09; 95% CI: −0.71 to 0.54) and then increased through 2020 (APC: 4.48; 95% CI: 1.22 to 7.85). In metropolitan areas, AAMR decreased from 1999 to 2003 (APC: −3.30; 95% CI: −5.35 to −1.20), remained relatively stable through 2015 (APC: 0.14; 95% CI: −0.30 to 0.58), and then increased through 2020 (APC: 3.00; 95% CI: 1.87 to 4.14) (Figure 4; Supplemental Tables 3 and 8).

**Fig. 4:**
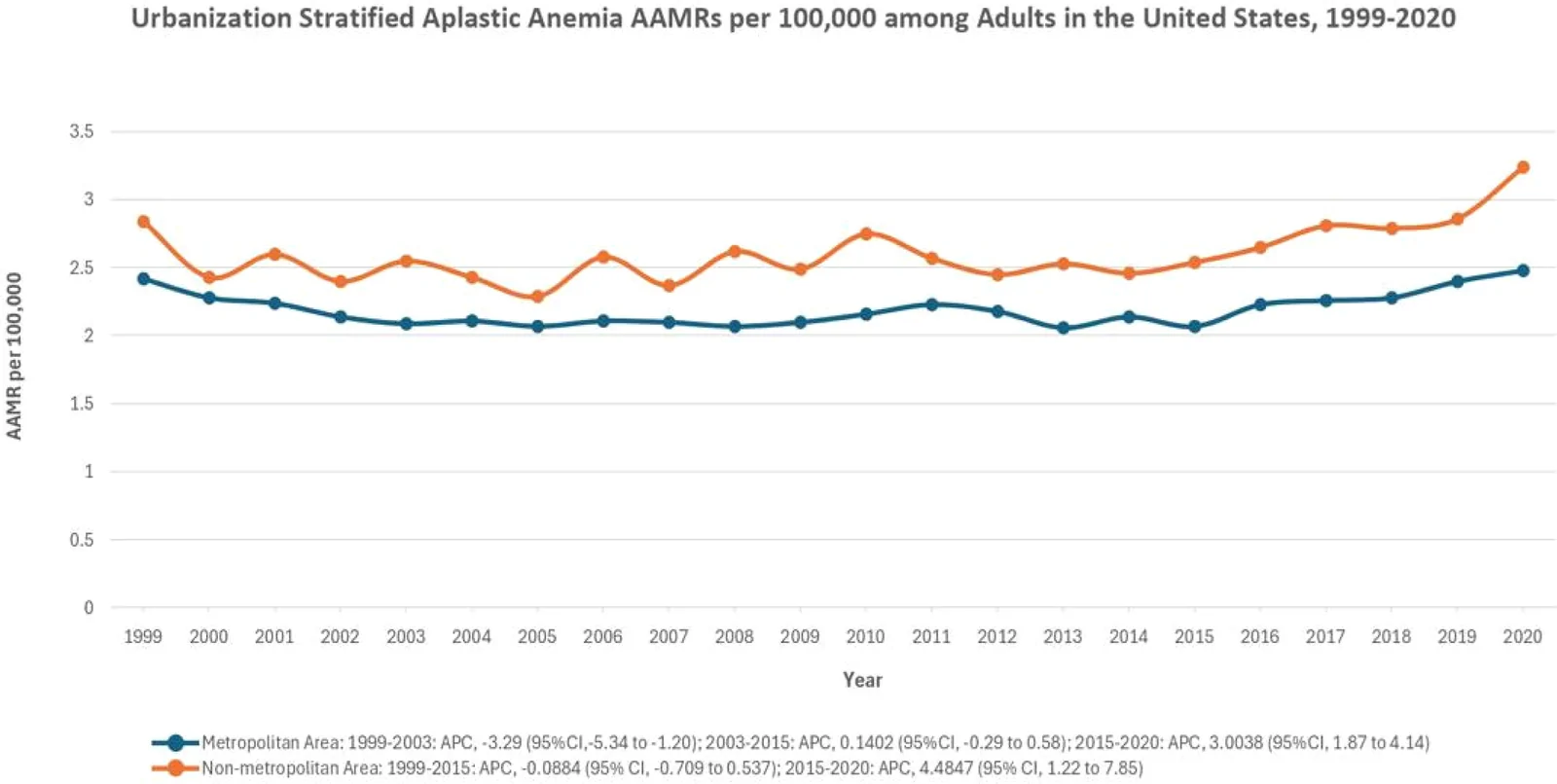
Age-adjusted mortality rates due to aplastic anemia among metropolitan and non-metropolitan US adults, 1999–2020.

## Intersectionality of the place of death and socioeconomic vulnerability

Place-of-death distribution differed significantly across combined sex and race/ethnicity strata (chi-square: p < 0.001), though the effect size was small (Cramér's V = 0.086). Medical facility deaths were more common among NH Black decedents, accounting for 77.3% of deaths among women and 78.0% among men, compared with NH White women (57.9%) and men (60.0%). Conversely, home/hospice deaths were more frequent among NH White men (25.1%) and women (21.8%) than among NH Black men (13.6%) and women (13.5%).

State-level social vulnerability was not positively associated with AAMR. Population-weighted overall SVI showed a non-significant inverse correlation with state-level AAMR (Pearson r = −0.23; p = 0.106; Spearman ρ = −0.17; p = 0.233), and theme-specific SVI analyzes did not support a positive association between state vulnerability and higher mortality rates.

Racial/ethnic mortality gaps similarly showed no significant association with state-level SVI. The Black to White AAMR difference (Pearson r = 0.20; p = 0.244; n = 36 states), Black to White AAMR ratio (Pearson r = 0.22; p = 0.195; n = 36), and the range between the highest and lowest race/ethnicity-specific AAMRs (Pearson r = −0.22; p = 0.156; n = 43) were not correlated with overall SVI (Table 1).

**Table 1:** Summary of intersectionality and socioeconomic vulnerability analyzes.

Analysis	Test	Result	Interpretation
Place of death by sex/race-ethnicity	Chi-square; Cramer’s V	p < 0.001; Cramer’s V = 0.086	Distribution differed significantly, but effect size was small
State AAMR vs overall SVI	Pearson and Spearman correlation	Pearson r = −0.23, p = 0.106; Spearman rho = −0.17, p = 0.233	No positive association
Black - White AAMR gap vs SVI	Pearson and Spearman correlation	Pearson r = 0.20, p = 0.244; Spearman rho = 0.261, p = 0.125	Not significant
Black - White AAMR ratio vs SVI	Pearson and Spearman correlation	Pearson r = 0.22, p = 0.195; Spearman rho = 0.287, p = 0.089	Not significant
Max - min race/ethnicity AAMR gap vs SVI	Pearson and Spearman correlation	Pearson r = −0.22, p = 0.156; Spearman rho = −0.13, p = 0.406	Not significant

AAMR: Age-adjusted mortality rates; SVI: Social Vulnerability Index.

## Discussion

This retrospective study provides a comprehensive analysis of trends in AA-related mortality in the US from 1999 to 2020, utilizing data from the CDC WONDER database. This study reveals notable variations across demographic and geographic categories, highlighting significant trends in mortality rates. First, the overall trend in the AAMR showed a steep decline through 2002, followed by a stable trend through 2015, and then a sharp increase in mortality through 2020. A similar pattern is seen in men and women, with men exhibiting an increased overall AA-related mortality compared to women. Second, NH African Americans/Blacks had the highest AAMR compared to the other racial groups. Third, noteworthy regional differences were also observed, with the Midwest showing the highest mortality rates, and states such as North Dakota having more than double the AAMR compared to other states such as New York. Moreover, non-metropolitan areas had significantly higher mortality rates than metropolitan areas. Understanding these patterns is crucial for developing targeted public health interventions to reduce mortality from AA. The key findings of this study are summarized in Figure 5.

**Fig. 5:**
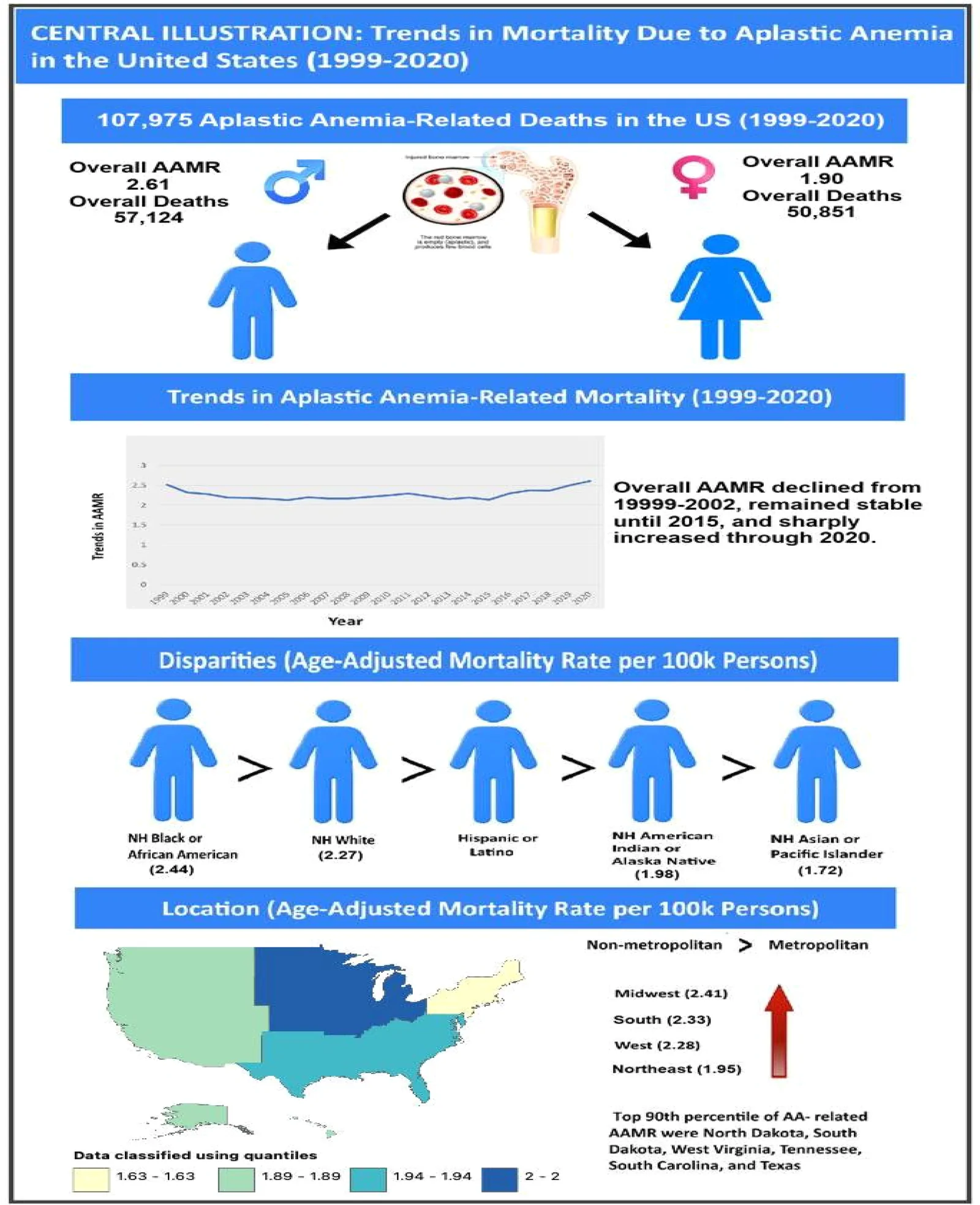
The key findings of this study.

The initial decline in AAMR from 1999 to 2002 followed by a stabilization of mortality rates through 2015, may be due to significant improvements in diagnosing and treating this disease, which was once considered uniformly fatal. Treatment modalities include allogeneic hematopoietic stem cell transplantation (HSCT), which is curative in 80% of children and 40% of adults, and immunosuppressive therapy (IST), with or without eltrombopag [2,5,15]. It has also been reported in previous studies that patients surviving five years following IST can expect survival similar to that of the general population [16]. Nevertheless, infection still remained an important cause of morbidity and mortality in patients who were unresponsive to HSCT and IST, particularly invasive fungal infections (IFIs). However, these patients have also had improvements in survival due to the decrease in infection-related mortality. These improved outcomes parallel advances in supportive care for infectious diseases, especially in antifungal therapy with voriconazole [17]. The steep increase in AAMR from 2015 to 2020 may be multifaceted and due to various reasons. AA has a peak incidence in the elderly population and is associated with worse mortality. In a recent international observational study, elderly patients with AA exhibited more severe thrombocytopenia, a lower response rate to first-line therapy, a higher incidence of evolution to myelodysplastic neoplasms, and inferior overall survival compared with younger patients. This finding, along with the fact that the US population is aging, could be a reason for the increased mortality observed after 2015 [18]. Furthermore, the long-term outcomes of HSCT and IST remain uncertain, particularly since both therapeutic interventions have been associated with the development of malignant neoplasms [16]. These findings may prove useful to practitioners, researchers, and policymakers for the effective mitigation of the burden due to this disease.

An upward trend was evident in both men and women, though men exhibited a more pronounced increase, consistent with previous epidemiological studies [7,19]. The persistent gender disparity in AA mortality, with higher AAMRs among men, aligns with prior research suggesting that biological and hormonal differences may influence disease susceptibility and progression [4]. Furthermore, exposure to specific chemicals like pesticides, insecticides, and benzene has been associated with the development of this condition. Men may experience higher exposure rates due to traditional occupational roles [20]. Another potential reason for the higher AAMR in men compared to women could be differences in health care utilization between the genders. Men might be less likely to seek timely medical attention, leading to poorer health outcomes and increased mortality [21].

The results of this study demonstrated significant differences in overall AAMR trends among racial groups. NH Black or African American and NH White populations exhibited significant changes over time, whereas Hispanic or Latino, NH American Indian or Alaska Native, and NH Asian or Pacific Islander populations showed overall stability in AAMR. NH Blacks or African Americans had the highest AAMR at nearly 1.5 times that of NH Asian or Pacific Islanders, who had the lowest. Initial significant decreases in AAMR were followed by subsequent increases among NH Black or African Americans and NH Whites. The initial decline in AAMR for these groups can be attributed to the implementation of the Affordable Care Act (ACA). Following the law’s enactment, the probability of being uninsured declined across all racial groups, with Black individuals experiencing a particularly notable decrease [22]. However, significant disparities persist, with NH Black or African Americans continuing to exhibit the highest AAMR. Several societal factors contribute to this disparity, including a higher prevalence of multimorbidity and worse average health status [23]. These disparities can, in part, be explained by racial differences in healthcare access, as demonstrated by Mahajan et al. [24]. Between 1999 and 2018, all racial groups except NH Blacks or African Americans and NH Whites showed a decline in the proportion of individuals without a usual care provider. Additionally, throughout this period, low-income NH Black or African American and NH White individuals were the most likely to forgo or delay medical care due to rising costs. In contrast, NH Asian or Pacific Islanders had the lowest likelihood of delaying or forgoing care due to financial constraints [24]. Further examinations of these disparities and the implementation of multidisciplinary interventions are crucial to mitigating these differences.

Place of death differed across sex and race/ethnicity groups, with NH Black adults more likely to die in a medical facility and less likely to die at home or in a hospice than NH White adults. While the overall association was modest, the pattern is consistent with well-documented disparities in hospice utilization and access to community-based end-of-life care among Black patients [25]. Whether this reflects differences in disease severity, referral practices, or care preferences cannot be determined from this data alone.

The SVI analysis yielded a somewhat unexpected finding: state-level social vulnerability did not correlate with higher AA mortality, nor did racial/ethnic mortality gaps appear to widen in more vulnerable states. This does not mean socioeconomic factors are unimportant; rather, it suggests that state-level SVI may be too broad a measure to capture the individual-level barriers most relevant to this disease. Because AA is a rare condition, clinical outcomes depend heavily on access to specialized tertiary centers, eligibility for hematopoietic cell transplantation, and prompt treatment initiation. Consequently, aggregated state-level indices are unlikely to accurately reflect these patient-level determinants of survival. Patient-level data on insurance status, proximity to transplant centers, and comorbidity burden will be needed to more precisely characterize the drivers of these disparities.

Geographically, the Midwest and South had the highest AAMRs, while the Northeast had the lowest. Regional differences may influence geographic disparities in AA mortality in healthcare infrastructure, socioeconomic factors, and environmental exposures. Both metropolitan and non-metropolitan areas showed an increasing trend in mortality throughout the study period. This contrasts with previous studies, which have shown an overall decrease in mortality from any cause in both regions from 1999 to 2019 [26]. The results of this study showed a higher overall mortality from AA in non-metropolitan areas than in metropolitan areas. This can be explained by the difficulty in accessing healthcare in these regions. This was illustrated by a report published by the American Hospital Association, which highlighted the closure of 136 hospitals between 2010 and 2020 across non-metropolitan America [27]. Furthermore, there has been a declining trend in the number of primary care physicians in non-metropolitan areas compared to metropolitan areas [28]. The massive decrease in primary care physician density and hospital closures can be explained by the declining population of non-metropolitan areas [29].

Results of this study that show an increasing AAMR from 2015 to 2020 should be interpreted with caution. They appear discordant with the Food and Drug Administration (FDA) approval of eltrombopag as frontline therapy in 2018, following the landmark randomized clinical trial demonstrating improved hematologic response rates [30]. However, several factors may explain these findings. First, there is an inherent lag between regulatory approval and widespread adoption. We think the mortality data from 2018–2020 likely reflect treatment patterns predating the broad implementation of eltrombopag. The year 2020 also coincided with the onset of the COVID-19 pandemic, which could have disproportionately affected immunocompromised patients. Finally, improvements in ICD-10 coding accuracy for rare bone marrow failure syndromes over time may have increased case capture, contributing to apparent rather than true increases in mortality, a form of surveillance bias that cannot be fully excluded using administrative data. However, data from a large US claims database showed that the incidence of AA, as captured by ICD-10 diagnosis codes, remained stable and even slightly declined from 2018 to 2022 (1.8 to 1.4 per million population), suggesting that improvements in diagnostic coding alone are unlikely to fully account for the observed increase in mortality [31].

## Strengths and limitations

The present study has several strengths, including the use of a large, nationally representative dataset and the application of robust statistical methods to analyze temporal trends. However, several limitations should be acknowledged. First, the reliance on death certificate data may introduce misclassification bias, as AA is often underdiagnosed or misdiagnosed. Second, the study did not account for individual-level factors, such as treatment history, comorbidities, or socioeconomic status, which may influence AA outcomes. Finally, the ecological nature of the study limits the ability to draw causal inferences.

## Conclusion

This study highlights significant temporal, demographic, and geographic disparities in AA-related mortality in the US from 1999 to 2020. The recent increase in mortality rates, particularly among men and rural populations, underscores the need for targeted public health interventions to address inequities in healthcare access and improve outcomes for AA patients. Future research should focus on identifying the drivers of these trends and evaluating strategies to reduce disparities, particularly in underserved communities. Addressing these challenges is essential to reducing the disease burden of AA and improving survival outcomes for affected populations.

## Funding information

No funding was received for the preparation of this article.

## Data availability

The data that support the findings of this study are available from the corresponding author upon reasonable request.

## Conflicts of interest

No conflict of interest to report.

## Appendix Supplementary materials

Image, application 1

## Footnotes

Supplementary material associated with this article can be found in the online version at doi:10.1016/j.htct.2026.108556.
