# Supplementary material for: Trends in mortality due to aplastic anemia in the United States, 1999-2020: a center for disease control wonder analysis
Source: Hematol Transfus Cell Ther. 2026 Nov 2;49(1):108556. doi: 10.1016/j.htct.2026.108556 (PMC13745042; doi:10.1016/j.htct.2026.108556)
Supplement: Supplementary file 1 [file mmc1.docx]

**Supplemental Table 1:** Overall Age-adjusted mortality rate due to aplastic anemia (1999–2020)

| **Year** | **Age Adjusted Rate** | **Age Adjusted Rate Lower 95% Confidence Interval** | **Age Adjusted Rate Upper 95% Confidence Interval** |
| --- | --- | --- | --- |
| 1999 | 2.51 | 2.43 | 2.58 |
| 2000 | 2.3 | 2.23 | 2.37 |
| 2001 | 2.28 | 2.21 | 2.35 |
| 2002 | 2.19 | 2.13 | 2.26 |
| 2003 | 2.17 | 2.11 | 2.24 |
| 2004 | 2.16 | 2.1 | 2.23 |
| 2005 | 2.13 | 2.07 | 2.2 |
| 2006 | 2.2 | 2.13 | 2.26 |
| 2007 | 2.17 | 2.1 | 2.23 |
| 2008 | 2.16 | 2.1 | 2.22 |
| 2009 | 2.18 | 2.12 | 2.25 |
| 2010 | 2.25 | 2.18 | 2.31 |
| 2011 | 2.29 | 2.23 | 2.36 |
| 2012 | 2.22 | 2.16 | 2.28 |
| 2013 | 2.11 | 2.05 | 2.17 |
| 2014 | 2.19 | 2.13 | 2.25 |
| 2015 | 2.14 | 2.08 | 2.2 |
| 2016 | 2.28 | 2.22 | 2.34 |
| 2017 | 2.37 | 2.31 | 2.43 |
| 2018 | 2.36 | 2.3 | 2.42 |
| 2019 | 2.47 | 2.41 | 2.54 |
| 2020 | 2.58 | 2.52 | 2.64 |
| Total | 2.27 | 2.26 | 2.28 |

**Supplemental Table 2:** Absolute number of deaths in adults with aplastic anemia stratified by place of death (1999–2020)

| **Year** | **Medical Facility** | **Decedent's home** | **Nursing/ Hospice facility** | **Other** |
| --- | --- | --- | --- | --- |
| 1999 | 3021 | 637 | 666 | 111 |
| 2000 | 2746 | 663 | 644 | 97 |
| 2001 | 2789 | 641 | 629 | 136 |
| 2002 | 2627 | 646 | 627 | 137 |
| 2003 | 2623 | 693 | 638 | 136 |
| 2004 | 2688 | 703 | 607 | 138 |
| 2005 | 2654 | 653 | 642 | 142 |
| 2006 | 2817 | 681 | 703 | 131 |
| 2007 | 2796 | 691 | 763 | 105 |
| 2008 | 2832 | 674 | 762 | 107 |
| 2009 | 2849 | 735 | 759 | 131 |
| 2010 | 3066 | 825 | 788 | 136 |
| 2011 | 3127 | 869 | 855 | 131 |
| 2012 | 3036 | 886 | 845 | 158 |
| 2013 | 3124 | 840 | 800 | 151 |
| 2014 | 3234 | 901 | 928 | 96 |
| 2015 | 3214 | 966 | 926 | 106 |
| 2016 | 3460 | 1056 | 997 | 111 |
| 2017 | 3527 | 1153 | 1093 | 137 |
| 2018 | 3609 | 1257 | 1091 | 134 |
| 2019 | 3885 | 1365 | 1076 | 128 |
| 2020 | 3969 | 1780 | 1060 | 157 |
| Total | 67693 | 19315 | 17899 | 2816 |

**Supplemental Table 3:** Annual percent change (APC) of aplastic anemia related to age-adjusted mortality rate (1999–2020)

| **Year interval** | **APC (95% CI)** |
| --- | --- |
| **Overall** |  |
| 1999 - 2002 | -4.22* (-8.03 – 0.25) |
| 2002 - 2015 | 0.08 ( -0.31 – 0.48) |
| 2015 - 2020 | 3.11* (1.73 – 4.50) |
| **Men** |  |
| 1999 - 2001 | -6.79 (-14.62 – 1.76) |
| 2001 - 2015 | -0.23 (-0.66 – 0.21) |
| 2015 - 2020 | 3.08* (1.49 – 4.69) |
| **Women** |  |
| 1999 - 2002 | -4.81*(-7.70 to -1.82) |
| 2002 - 2015 | 0.16 ( -0.24 – 0.57) |
| 2015 - 2020 | 3.36* (1.96 – 4.79) |
| **Hispanic or Latino** |  |
| 1999 - 2020 | 0.28 (-0.07 – 0.63) |
| **American Indian or Alaskan Natives** |  |
| 1999 - 2020 | 0.11 (-1.05 – 1.29) |
| **Black or African Americans** |  |
| 1999 - 2016 | -1.21* (-1.72 to -0.69) |
| 2016 - 2020 | 4.71* (0.50 – 9.10) |
| **Whites** |  |
| 1999 - 2002 | -4.09* (-7.86 to -0.17) |
| 2002 - 2015 | 0.26 (- 0.18 – 0.71) |
| 2015 - 2020 | 3.29* (1.97 – 4.62) |
| **Metropolitan Areas** |  |
| 1999 - 2003 | -3.30* (-5.35 to -1.20) |
| 2003 - 2015 | 0.14 (-0.30 – 0.58) |
| 2015 - 2020 | 3.00* (1.87 – 4.14) |
| **Non-Metropolitan Areas** |  |
| 1999 - 2015 | -0.09 (-0.71 – 0.54) |
| 2015 - 2020 | 4.48* (1.22 – 7.85) |

**Supplemental Table 4:** Age-adjusted mortality rate due to aplastic anemia stratified by sex (1999–2020)

| **Sex** | **Year** | **Age Adjusted Rate** | **Age Adjusted Rate Lower 95% Confidence Interval** | **Age Adjusted Rate Upper 95% Confidence Interval** |
| --- | --- | --- | --- | --- |
| Female | 1999 | 2.04 | 1.95 | 2.13 |
| Female | 2000 | 2 | 1.92 | 2.09 |
| Female | 2001 | 1.98 | 1.89 | 2.07 |
| Female | 2002 | 1.74 | 1.66 | 1.82 |
| Female | 2003 | 1.76 | 1.68 | 1.84 |
| Female | 2004 | 1.81 | 1.73 | 1.89 |
| Female | 2005 | 1.75 | 1.67 | 1.83 |
| Female | 2006 | 1.86 | 1.78 | 1.93 |
| Female | 2007 | 1.82 | 1.74 | 1.9 |
| Female | 2008 | 1.81 | 1.73 | 1.89 |
| Female | 2009 | 1.8 | 1.72 | 1.87 |
| Female | 2010 | 1.84 | 1.76 | 1.92 |
| Female | 2011 | 1.85 | 1.77 | 1.93 |
| Female | 2012 | 1.86 | 1.78 | 1.94 |
| Female | 2013 | 1.75 | 1.68 | 1.83 |
| Female | 2014 | 1.86 | 1.78 | 1.94 |
| Female | 2015 | 1.8 | 1.73 | 1.87 |
| Female | 2016 | 1.88 | 1.81 | 1.95 |
| Female | 2017 | 2.03 | 1.95 | 2.1 |
| Female | 2018 | 2.02 | 1.94 | 2.09 |
| Female | 2019 | 2.05 | 1.97 | 2.13 |
| Female | 2020 | 2.18 | 2.11 | 2.26 |
| Male | 1999 | 3.27 | 3.14 | 3.41 |
| Male | 2000 | 2.83 | 2.7 | 2.95 |
| Male | 2001 | 2.82 | 2.7 | 2.95 |
| Male | 2002 | 2.84 | 2.72 | 2.97 |
| Male | 2003 | 2.79 | 2.67 | 2.91 |
| Male | 2004 | 2.73 | 2.61 | 2.84 |
| Male | 2005 | 2.64 | 2.52 | 2.75 |
| Male | 2006 | 2.7 | 2.58 | 2.81 |
| Male | 2007 | 2.68 | 2.57 | 2.79 |
| Male | 2008 | 2.65 | 2.54 | 2.76 |
| Male | 2009 | 2.75 | 2.64 | 2.86 |
| Male | 2010 | 2.85 | 2.74 | 2.97 |
| Male | 2011 | 2.86 | 2.75 | 2.97 |
| Male | 2012 | 2.66 | 2.56 | 2.77 |
| Male | 2013 | 2.7 | 2.6 | 2.81 |
| Male | 2014 | 2.67 | 2.57 | 2.77 |
| Male | 2015 | 2.64 | 2.54 | 2.74 |
| Male | 2016 | 2.82 | 2.72 | 2.92 |
| Male | 2017 | 2.83 | 2.73 | 2.94 |
| Male | 2018 | 2.85 | 2.75 | 2.95 |
| Male | 2019 | 3.04 | 2.93 | 3.14 |
| Male | 2020 | 3.16 | 3.06 | 3.27 |

**Supplemental Table 5:** Age-adjusted mortality rate due to aplastic anemia stratified by race (1999–2020)

| **Race** | **Year** | **Age Adjusted Rate** | **Age Adjusted Rate Lower 95% Confidence Interval** | **Age Adjusted Rate Upper 95% Confidence Interval** |
| --- | --- | --- | --- | --- |
| American Indian or Alaska Native | 1999 | 2.05 | 1.21 | 3.24 |
| American Indian or Alaska Native | 2000 | 2.01 | 1.19 | 3.17 |
| American Indian or Alaska Native | 2001 | 2.53 | 1.58 | 3.83 |
| American Indian or Alaska Native | 2003 | 2.13 | 1.32 | 3.26 |
| American Indian or Alaska Native | 2004 | 2.59 | 1.62 | 3.93 |
| American Indian or Alaska Native | 2005 | 1.91 | 1.18 | 2.92 |
| American Indian or Alaska Native | 2006 | 2.04 | 1.28 | 3.09 |
| American Indian or Alaska Native | 2007 | 1.78 | 1.05 | 2.81 |
| American Indian or Alaska Native | 2008 | 1.81 | 1.15 | 2.71 |
| American Indian or Alaska Native | 2009 | 1.59 | 0.99 | 2.43 |
| American Indian or Alaska Native | 2010 | 2.02 | 1.3 | 3.01 |
| American Indian or Alaska Native | 2011 | 1.37 | 0.84 | 2.12 |
| American Indian or Alaska Native | 2012 | 1.76 | 1.17 | 2.55 |
| American Indian or Alaska Native | 2013 | 2.01 | 1.4 | 2.8 |
| American Indian or Alaska Native | 2014 | 2.41 | 1.72 | 3.28 |
| American Indian or Alaska Native | 2015 | 2.54 | 1.86 | 3.38 |
| American Indian or Alaska Native | 2016 | 1.75 | 1.24 | 2.39 |
| American Indian or Alaska Native | 2017 | 2.01 | 1.46 | 2.69 |
| American Indian or Alaska Native | 2018 | 1.91 | 1.36 | 2.6 |
| American Indian or Alaska Native | 2019 | 2.44 | 1.83 | 3.19 |
| American Indian or Alaska Native | 2020 | 2.07 | 1.54 | 2.72 |
| Asian or Pacific Islander | 1999 | 2.26 | 1.79 | 2.74 |
| Asian or Pacific Islander | 2000 | 2.08 | 1.67 | 2.55 |
| Asian or Pacific Islander | 2001 | 1.93 | 1.55 | 2.37 |
| Asian or Pacific Islander | 2002 | 1.6 | 1.27 | 1.98 |
| Asian or Pacific Islander | 2003 | 1.63 | 1.3 | 2.02 |
| Asian or Pacific Islander | 2004 | 2.26 | 1.86 | 2.66 |
| Asian or Pacific Islander | 2005 | 1.73 | 1.39 | 2.07 |
| Asian or Pacific Islander | 2006 | 1.6 | 1.29 | 1.92 |
| Asian or Pacific Islander | 2007 | 1.9 | 1.56 | 2.24 |
| Asian or Pacific Islander | 2008 | 1.62 | 1.32 | 1.91 |
| Asian or Pacific Islander | 2009 | 1.68 | 1.38 | 1.98 |
| Asian or Pacific Islander | 2010 | 1.77 | 1.47 | 2.07 |
| Asian or Pacific Islander | 2011 | 1.73 | 1.44 | 2.01 |
| Asian or Pacific Islander | 2012 | 1.83 | 1.55 | 2.11 |
| Asian or Pacific Islander | 2013 | 1.61 | 1.36 | 1.87 |
| Asian or Pacific Islander | 2014 | 1.53 | 1.29 | 1.77 |
| Asian or Pacific Islander | 2015 | 1.71 | 1.46 | 1.95 |
| Asian or Pacific Islander | 2016 | 1.47 | 1.25 | 1.69 |
| Asian or Pacific Islander | 2017 | 1.79 | 1.55 | 2.03 |
| Asian or Pacific Islander | 2018 | 1.8 | 1.56 | 2.03 |
| Asian or Pacific Islander | 2019 | 1.86 | 1.62 | 2.09 |
| Asian or Pacific Islander | 2020 | 1.68 | 1.47 | 1.9 |
| Black or African American | 1999 | 2.98 | 2.71 | 3.25 |
| Black or African American | 2000 | 2.63 | 2.38 | 2.88 |
| Black or African American | 2001 | 2.55 | 2.31 | 2.8 |
| Black or African American | 2002 | 2.55 | 2.3 | 2.79 |
| Black or African American | 2003 | 2.52 | 2.28 | 2.76 |
| Black or African American | 2004 | 2.59 | 2.34 | 2.83 |
| Black or African American | 2005 | 2.2 | 1.99 | 2.42 |
| Black or African American | 2006 | 2.45 | 2.22 | 2.68 |
| Black or African American | 2007 | 2.43 | 2.21 | 2.66 |
| Black or African American | 2008 | 2.29 | 2.07 | 2.5 |
| Black or African American | 2009 | 2.35 | 2.14 | 2.57 |
| Black or African American | 2010 | 2.36 | 2.15 | 2.58 |
| Black or African American | 2011 | 2.5 | 2.28 | 2.71 |
| Black or African American | 2012 | 2.41 | 2.2 | 2.62 |
| Black or African American | 2013 | 2.21 | 2.01 | 2.41 |
| Black or African American | 2014 | 2.27 | 2.07 | 2.47 |
| Black or African American | 2015 | 2.11 | 1.92 | 2.29 |
| Black or African American | 2016 | 2.26 | 2.07 | 2.45 |
| Black or African American | 2017 | 2.33 | 2.14 | 2.52 |
| Black or African American | 2018 | 2.31 | 2.13 | 2.5 |
| Black or African American | 2019 | 2.52 | 2.33 | 2.71 |
| Black or African American | 2020 | 2.65 | 2.46 | 2.84 |
| White | 1999 | 2.45 | 2.37 | 2.53 |
| White | 2000 | 2.26 | 2.19 | 2.34 |
| White | 2001 | 2.27 | 2.19 | 2.34 |
| White | 2002 | 2.15 | 2.08 | 2.23 |
| White | 2003 | 2.17 | 2.1 | 2.24 |
| White | 2004 | 2.14 | 2.07 | 2.21 |
| White | 2005 | 2.1 | 2.03 | 2.17 |
| White | 2006 | 2.18 | 2.11 | 2.25 |
| White | 2007 | 2.13 | 2.06 | 2.2 |
| White | 2008 | 2.15 | 2.08 | 2.22 |
| White | 2009 | 2.18 | 2.11 | 2.24 |
| White | 2010 | 2.28 | 2.21 | 2.35 |
| White | 2011 | 2.26 | 2.2 | 2.33 |
| White | 2012 | 2.16 | 2.09 | 2.23 |
| White | 2013 | 2.13 | 2.07 | 2.2 |
| White | 2014 | 2.22 | 2.15 | 2.29 |
| White | 2015 | 2.18 | 2.12 | 2.25 |
| White | 2016 | 2.34 | 2.27 | 2.41 |
| White | 2017 | 2.39 | 2.32 | 2.45 |
| White | 2018 | 2.4 | 2.33 | 2.47 |
| White | 2019 | 2.48 | 2.41 | 2.55 |
| White | 2020 | 2.63 | 2.57 | 2.7 |
| Hispanic or Latino | 1999 | 2.2 | 1.89 | 2.51 |
| Hispanic or Latino | 2000 | 2.01 | 1.72 | 2.29 |
| Hispanic or Latino | 2001 | 2.2 | 1.9 | 2.5 |
| Hispanic or Latino | 2002 | 2.15 | 1.86 | 2.43 |
| Hispanic or Latino | 2003 | 1.84 | 1.59 | 2.1 |
| Hispanic or Latino | 2004 | 1.88 | 1.63 | 2.13 |
| Hispanic or Latino | 2005 | 1.98 | 1.72 | 2.23 |
| Hispanic or Latino | 2006 | 2.12 | 1.87 | 2.38 |
| Hispanic or Latino | 2007 | 1.97 | 1.73 | 2.21 |
| Hispanic or Latino | 2008 | 1.91 | 1.69 | 2.14 |
| Hispanic or Latino | 2009 | 2.04 | 1.81 | 2.27 |
| Hispanic or Latino | 2010 | 2.03 | 1.81 | 2.26 |
| Hispanic or Latino | 2011 | 2.04 | 1.83 | 2.26 |
| Hispanic or Latino | 2012 | 2.04 | 1.83 | 2.25 |
| Hispanic or Latino | 2013 | 2 | 1.8 | 2.2 |
| Hispanic or Latino | 2014 | 2.02 | 1.83 | 2.22 |
| Hispanic or Latino | 2015 | 1.94 | 1.75 | 2.13 |
| Hispanic or Latino | 2016 | 2.02 | 1.83 | 2.21 |
| Hispanic or Latino | 2017 | 2.21 | 2.02 | 2.4 |
| Hispanic or Latino | 2018 | 2.1 | 1.92 | 2.28 |
| Hispanic or Latino | 2019 | 2.12 | 1.94 | 2.3 |
| Hispanic or Latino | 2020 | 2.23 | 2.05 | 2.41 |

**Supplemental Table 6:** Age-adjusted mortality rate due to aplastic anemia, stratified by states

| **State** | **Age Adjusted Rate** | **Age Adjusted Rate Lower 95% Confidence Interval** | **Age Adjusted Rate Upper 95% Confidence Interval** |
| --- | --- | --- | --- |
| Alabama | 2.54 | 2.42 | 2.65 |
| Alaska | 2.51 | 2.11 | 2.9 |
| Arizona | 2.04 | 1.95 | 2.13 |
| Arkansas | 2.43 | 2.28 | 2.57 |
| California | 2.36 | 2.32 | 2.4 |
| Colorado | 1.92 | 1.81 | 2.03 |
| Connecticut | 1.86 | 1.75 | 1.97 |
| Delaware | 2.19 | 1.95 | 2.43 |
| District of Columbia | 2.51 | 2.17 | 2.85 |
| Florida | 2.01 | 1.96 | 2.05 |
| Georgia | 1.9 | 1.82 | 1.97 |
| Hawaii | 2.69 | 2.47 | 2.9 |
| Idaho | 2.16 | 1.97 | 2.35 |
| Illinois | 2.14 | 2.07 | 2.21 |
| Indiana | 2.63 | 2.53 | 2.73 |
| Iowa | 2.49 | 2.35 | 2.62 |
| Kansas | 2.7 | 2.55 | 2.85 |
| Kentucky | 2.76 | 2.64 | 2.89 |
| Louisiana | 1.7 | 1.6 | 1.8 |
| Maine | 2.39 | 2.2 | 2.59 |
| Maryland | 2.55 | 2.44 | 2.66 |
| Massachusetts | 1.66 | 1.58 | 1.74 |
| Michigan | 2.13 | 2.06 | 2.2 |
| Minnesota | 2.75 | 2.64 | 2.86 |
| Mississippi | 2.09 | 1.96 | 2.23 |
| Missouri | 2.27 | 2.17 | 2.36 |
| Montana | 2.06 | 1.84 | 2.28 |
| Nebraska | 2.65 | 2.46 | 2.84 |
| Nevada | 1.84 | 1.7 | 1.98 |
| New Hampshire | 1.94 | 1.75 | 2.13 |
| New Jersey | 2.24 | 2.16 | 2.32 |
| New Mexico | 1.82 | 1.67 | 1.98 |
| New York | 1.49 | 1.45 | 1.53 |
| North Carolina | 2.31 | 2.23 | 2.39 |
| North Dakota | 3.32 | 2.99 | 3.66 |
| Ohio | 2.57 | 2.49 | 2.64 |
| Oklahoma | 2.32 | 2.19 | 2.44 |
| Oregon | 2.26 | 2.14 | 2.38 |
| Pennsylvania | 2.45 | 2.39 | 2.51 |
| Rhode Island | 2.41 | 2.19 | 2.64 |
| South Carolina | 2.74 | 2.62 | 2.87 |
| South Dakota | 3.2 | 2.9 | 3.5 |
| Tennessee | 2.82 | 2.71 | 2.92 |
| Texas | 2.78 | 2.72 | 2.84 |
| Utah | 2.02 | 1.86 | 2.18 |
| Vermont | 2.32 | 2.02 | 2.61 |
| Virginia | 1.89 | 1.81 | 1.97 |
| Washington | 2.71 | 2.6 | 2.81 |
| West Virginia | 3.1 | 2.91 | 3.29 |
| Wisconsin | 2.13 | 2.04 | 2.23 |
| Wyoming | 2.08 | 1.76 | 2.39 |

**Supplemental Table 7:** Age-adjusted mortality rate due to aplastic anemia stratified by census region (1999–2020)

| **Census Region** | **Year** | **Age Adjusted Rate** | **Age Adjusted Rate Lower 95% Confidence Interval** | **Age Adjusted Rate Upper 95% Confidence Interval** |
| --- | --- | --- | --- | --- |
| Census Region 1: Northeast | 1999 | 2.41 | 2.26 | 2.57 |
| Census Region 1: Northeast | 2000 | 2.23 | 2.07 | 2.38 |
| Census Region 1: Northeast | 2001 | 2.21 | 2.06 | 2.36 |
| Census Region 1: Northeast | 2002 | 1.98 | 1.84 | 2.12 |
| Census Region 1: Northeast | 2003 | 1.94 | 1.8 | 2.08 |
| Census Region 1: Northeast | 2004 | 1.92 | 1.78 | 2.06 |
| Census Region 1: Northeast | 2005 | 1.9 | 1.77 | 2.04 |
| Census Region 1: Northeast | 2006 | 1.77 | 1.64 | 1.9 |
| Census Region 1: Northeast | 2007 | 1.95 | 1.82 | 2.09 |
| Census Region 1: Northeast | 2008 | 1.84 | 1.71 | 1.97 |
| Census Region 1: Northeast | 2009 | 1.86 | 1.73 | 1.99 |
| Census Region 1: Northeast | 2010 | 1.86 | 1.73 | 1.99 |
| Census Region 1: Northeast | 2011 | 2.04 | 1.9 | 2.18 |
| Census Region 1: Northeast | 2012 | 1.95 | 1.82 | 2.09 |
| Census Region 1: Northeast | 2013 | 1.84 | 1.71 | 1.97 |
| Census Region 1: Northeast | 2014 | 1.88 | 1.76 | 2.01 |
| Census Region 1: Northeast | 2015 | 1.81 | 1.69 | 1.94 |
| Census Region 1: Northeast | 2016 | 1.83 | 1.71 | 1.96 |
| Census Region 1: Northeast | 2017 | 1.92 | 1.79 | 2.05 |
| Census Region 1: Northeast | 2018 | 1.91 | 1.79 | 2.04 |
| Census Region 1: Northeast | 2019 | 1.9 | 1.78 | 2.03 |
| Census Region 1: Northeast | 2020 | 2.13 | 1.99 | 2.26 |
| Census Region 2: Midwest | 1999 | 2.67 | 2.51 | 2.83 |
| Census Region 2: Midwest | 2000 | 2.41 | 2.26 | 2.56 |
| Census Region 2: Midwest | 2001 | 2.52 | 2.36 | 2.67 |
| Census Region 2: Midwest | 2002 | 2.21 | 2.07 | 2.35 |
| Census Region 2: Midwest | 2003 | 2.44 | 2.3 | 2.59 |
| Census Region 2: Midwest | 2004 | 2.25 | 2.11 | 2.39 |
| Census Region 2: Midwest | 2005 | 2.2 | 2.06 | 2.34 |
| Census Region 2: Midwest | 2006 | 2.35 | 2.2 | 2.49 |
| Census Region 2: Midwest | 2007 | 2.35 | 2.21 | 2.49 |
| Census Region 2: Midwest | 2008 | 2.31 | 2.17 | 2.45 |
| Census Region 2: Midwest | 2009 | 2.39 | 2.25 | 2.53 |
| Census Region 2: Midwest | 2010 | 2.4 | 2.26 | 2.54 |
| Census Region 2: Midwest | 2011 | 2.37 | 2.23 | 2.51 |
| Census Region 2: Midwest | 2012 | 2.28 | 2.15 | 2.42 |
| Census Region 2: Midwest | 2013 | 2.15 | 2.02 | 2.28 |
| Census Region 2: Midwest | 2014 | 2.27 | 2.14 | 2.4 |
| Census Region 2: Midwest | 2015 | 2.25 | 2.12 | 2.38 |
| Census Region 2: Midwest | 2016 | 2.44 | 2.31 | 2.58 |
| Census Region 2: Midwest | 2017 | 2.51 | 2.37 | 2.64 |
| Census Region 2: Midwest | 2018 | 2.46 | 2.32 | 2.59 |
| Census Region 2: Midwest | 2019 | 2.68 | 2.55 | 2.82 |
| Census Region 2: Midwest | 2020 | 2.85 | 2.71 | 2.99 |
| Census Region 3: South | 1999 | 2.45 | 2.33 | 2.57 |
| Census Region 3: South | 2000 | 2.36 | 2.24 | 2.48 |
| Census Region 3: South | 2001 | 2.31 | 2.19 | 2.42 |
| Census Region 3: South | 2002 | 2.24 | 2.12 | 2.35 |
| Census Region 3: South | 2003 | 2.18 | 2.07 | 2.3 |
| Census Region 3: South | 2004 | 2.29 | 2.18 | 2.41 |
| Census Region 3: South | 2005 | 2.2 | 2.09 | 2.31 |
| Census Region 3: South | 2006 | 2.28 | 2.17 | 2.4 |
| Census Region 3: South | 2007 | 2.18 | 2.07 | 2.29 |
| Census Region 3: South | 2008 | 2.23 | 2.12 | 2.33 |
| Census Region 3: South | 2009 | 2.26 | 2.15 | 2.37 |
| Census Region 3: South | 2010 | 2.36 | 2.25 | 2.47 |
| Census Region 3: South | 2011 | 2.34 | 2.24 | 2.45 |
| Census Region 3: South | 2012 | 2.25 | 2.14 | 2.35 |
| Census Region 3: South | 2013 | 2.29 | 2.19 | 2.4 |
| Census Region 3: South | 2014 | 2.32 | 2.22 | 2.42 |
| Census Region 3: South | 2015 | 2.18 | 2.09 | 2.28 |
| Census Region 3: South | 2016 | 2.4 | 2.3 | 2.5 |
| Census Region 3: South | 2017 | 2.38 | 2.28 | 2.48 |
| Census Region 3: South | 2018 | 2.48 | 2.38 | 2.58 |
| Census Region 3: South | 2019 | 2.51 | 2.41 | 2.61 |
| Census Region 3: South | 2020 | 2.7 | 2.59 | 2.8 |
| Census Region 4: West | 1999 | 2.53 | 2.37 | 2.7 |
| Census Region 4: West | 2000 | 2.23 | 2.08 | 2.39 |
| Census Region 4: West | 2001 | 2.14 | 1.99 | 2.29 |
| Census Region 4: West | 2002 | 2.27 | 2.12 | 2.43 |
| Census Region 4: West | 2003 | 2.14 | 2 | 2.29 |
| Census Region 4: West | 2004 | 2.13 | 1.99 | 2.27 |
| Census Region 4: West | 2005 | 2.05 | 1.91 | 2.19 |
| Census Region 4: West | 2006 | 2.25 | 2.1 | 2.39 |
| Census Region 4: West | 2007 | 2.1 | 1.96 | 2.24 |
| Census Region 4: West | 2008 | 2.14 | 2 | 2.28 |
| Census Region 4: West | 2009 | 2.1 | 1.96 | 2.24 |
| Census Region 4: West | 2010 | 2.28 | 2.14 | 2.41 |
| Census Region 4: West | 2011 | 2.21 | 2.08 | 2.35 |
| Census Region 4: West | 2012 | 2.17 | 2.04 | 2.3 |
| Census Region 4: West | 2013 | 2.12 | 1.99 | 2.24 |
| Census Region 4: West | 2014 | 2.17 | 2.04 | 2.3 |
| Census Region 4: West | 2015 | 2.29 | 2.16 | 2.42 |
| Census Region 4: West | 2016 | 2.24 | 2.11 | 2.37 |
| Census Region 4: West | 2017 | 2.5 | 2.36 | 2.63 |
| Census Region 4: West | 2018 | 2.45 | 2.32 | 2.58 |
| Census Region 4: West | 2019 | 2.63 | 2.5 | 2.77 |
| Census Region 4: West | 2020 | 2.59 | 2.46 | 2.72 |

**Supplemental Table 8: Age-adjusted mortality rate due to aplastic anemia stratified by urbanization (1999–2020)**

| **Urbanization** | **Year** | **Age Adjusted Rate** | **Age Adjusted Rate Lower 95% Confidence Interval** | **Age Adjusted Rate Upper 95% Confidence Interval** |
| --- | --- | --- | --- | --- |
| Metropolitan | 1999 | 2.42 | 2.34 | 2.5 |
| Metropolitan | 2000 | 2.28 | 2.2 | 2.36 |
| Metropolitan | 2001 | 2.24 | 2.16 | 2.31 |
| Metropolitan | 2002 | 2.14 | 2.07 | 2.22 |
| Metropolitan | 2003 | 2.09 | 2.02 | 2.17 |
| Metropolitan | 2004 | 2.11 | 2.04 | 2.18 |
| Metropolitan | 2005 | 2.07 | 2 | 2.14 |
| Metropolitan | 2006 | 2.11 | 2.03 | 2.18 |
| Metropolitan | 2007 | 2.1 | 2.03 | 2.17 |
| Metropolitan | 2008 | 2.07 | 2 | 2.14 |
| Metropolitan | 2009 | 2.1 | 2.03 | 2.17 |
| Metropolitan | 2010 | 2.16 | 2.09 | 2.23 |
| Metropolitan | 2011 | 2.23 | 2.16 | 2.3 |
| Metropolitan | 2012 | 2.18 | 2.11 | 2.25 |
| Metropolitan | 2013 | 2.06 | 1.99 | 2.12 |
| Metropolitan | 2014 | 2.14 | 2.07 | 2.21 |
| Metropolitan | 2015 | 2.07 | 2 | 2.13 |
| Metropolitan | 2016 | 2.23 | 2.16 | 2.29 |
| Metropolitan | 2017 | 2.26 | 2.19 | 2.32 |
| Metropolitan | 2018 | 2.28 | 2.22 | 2.35 |
| Metropolitan | 2019 | 2.4 | 2.33 | 2.47 |
| Metropolitan | 2020 | 2.48 | 2.41 | 2.54 |
| Non-metropolitan | 1999 | 2.84 | 2.66 | 3.02 |
| Non-metropolitan | 2000 | 2.43 | 2.26 | 2.6 |
| Non-metropolitan | 2001 | 2.6 | 2.43 | 2.77 |
| Non-metropolitan | 2002 | 2.4 | 2.23 | 2.56 |
| Non-metropolitan | 2003 | 2.55 | 2.38 | 2.72 |
| Non-metropolitan | 2004 | 2.43 | 2.26 | 2.59 |
| Non-metropolitan | 2005 | 2.29 | 2.13 | 2.45 |
| Non-metropolitan | 2006 | 2.58 | 2.41 | 2.75 |
| Non-metropolitan | 2007 | 2.37 | 2.21 | 2.53 |
| Non-metropolitan | 2008 | 2.62 | 2.46 | 2.79 |
| Non-metropolitan | 2009 | 2.49 | 2.33 | 2.65 |
| Non-metropolitan | 2010 | 2.75 | 2.58 | 2.92 |
| Non-metropolitan | 2011 | 2.57 | 2.41 | 2.73 |
| Non-metropolitan | 2012 | 2.45 | 2.29 | 2.61 |
| Non-metropolitan | 2013 | 2.53 | 2.36 | 2.69 |
| Non-metropolitan | 2014 | 2.46 | 2.3 | 2.62 |
| Non-metropolitan | 2015 | 2.54 | 2.38 | 2.7 |
| Non-metropolitan | 2016 | 2.65 | 2.49 | 2.81 |
| Non-metropolitan | 2017 | 2.81 | 2.64 | 2.97 |
| Non-metropolitan | 2018 | 2.79 | 2.62 | 2.95 |
| Non-metropolitan | 2019 | 2.86 | 2.69 | 3.02 |
| Non-metropolitan | 2020 | 3.24 | 3.06 | 3.42 |
